# Comparing the Use of an Online Expert Health Network against Common Information Sources to Answer Health Questions

**DOI:** 10.2196/jmir.1886

**Published:** 2012-02-02

**Authors:** Martijn DF Rhebergen, Annet F Lenderink, Frank JH van Dijk, Carel TJ Hulshof

**Affiliations:** ^1^Academic Medical Center AmsterdamCoronel Institute of Occupational HealthUniversity of AmsterdamAmsterdamNetherlands; ^2^Academic Medical Center AmsterdamDutch Center for Occupational DiseasesUniversity of AmsterdamAmsterdamNetherlands; ^3^Netherlands Society of Occupational Medicine (NVAB)Centre of ExcellenceUtrechtNetherlands

**Keywords:** Information services, online expert network, medical informatics, information-seeking behavior, occupational health, evidence-based practice, question and answer

## Abstract

**Background:**

Many workers have questions about occupational safety and health (OSH). It is unknown whether workers are able to find correct, evidence-based answers to OSH questions when they use common information sources, such as websites, or whether they would benefit from using an easily accessible, free-of-charge online network of OSH experts providing advice.

**Objective:**

To assess the rate of correct, evidence-based answers to OSH questions in a group of workers who used an online network of OSH experts (intervention group) compared with a group of workers who used common information sources (control group).

**Methods:**

In a quasi-experimental study, workers in the intervention and control groups were randomly offered 2 questions from a pool of 16 standardized OSH questions. Both questions were sent by mail to all participants, who had 3 weeks to answer them. The intervention group was instructed to use only the online network ArboAntwoord, a network of about 80 OSH experts, to solve the questions. The control group was instructed that they could use all information sources available to them. To assess answer correctness as the main study outcome, 16 standardized correct model answers were constructed with the help of reviewers who performed literature searches. Subsequently, the answers provided by all participants in the intervention (n = 94 answers) and control groups (n = 124 answers) were blinded and compared with the correct model answers on the degree of correctness.

**Results:**

Of the 94 answers given by participants in the intervention group, 58 were correct (62%), compared with 24 of the 124 answers (19%) in the control group, who mainly used informational websites found via Google. The difference between the 2 groups was significant (rate difference = 43%, 95% confidence interval [CI] 30%–54%). Additional analysis showed that the rate of correct main conclusions of the answers was 85 of 94 answers (90%) in the intervention group and 75 of 124 answers (61%) in the control group (rate difference = 29%, 95% CI 19%–40%). Remarkably, we could not identify differences between workers who provided correct answers and workers who did not on how they experienced the credibility, completeness, and applicability of the information found (*P* > .05).

**Conclusions:**

Workers are often unable to find correct answers to OSH questions when using common information sources, generally informational websites. Because workers frequently misjudge the quality of the information they find, other strategies are required to assist workers in finding correct answers. Expert advice provided through an online expert network can be effective for this purpose. As many people experience difficulties in finding correct answers to their health questions, expert networks may be an attractive new source of information for health fields in general.

## Introduction

Many workers seek information to answer occupational safety and health (OSH) questions [1-4]. For this purpose, workers commonly use a variety of sources, such as asking advice from OSH experts within their company or social network, or exploring informational websites [5-7]. Ideally, the information available from these common information sources is of high quality [6,7], as low-quality information may lead to incorrect answers and wrong decisions regarding the prevention or management of OSH at work [8]. It is unknown whether workers can find correct, evidence-based answers [9,10].

In general, one might ask whether it is possible for workers to find correct answers to their OSH questions. Finding information and answering health-related questions require specific skills or literacy [11,12]. The World Health Organization defines health literacy as “the cognitive and social skills which determine the motivation and the ability of an individual to gain access to, understand and use information in ways that promote and maintain good health” [13]. The Internet, the source that workers use most frequently, often provides excessive amounts of information that is not always easy to understand or of high quality [14-17]. The solution might be to consult OSH experts who are trained in finding evidence-based answers to clinical questions [18,19]. However, the consultation of experts by workers might be hampered by restricted access and high costs [20]. An attractive solution might be a selection of the best of both options by offering workers easily accessible, free-of-charge online advice from OSH experts [21-23].

Question-and-answer expert network tools could be useful for providing such online expert advice, as these tools are designed for communication, and knowledge dissemination, storage, and retrieval [22,23]. These tools have the potential to build a network of experts on a particular topic (and many subtopics) and make them accessible to questioners. We tested the hypothesis that workers who used this online network of OSH experts would find correct answers to OSH questions more often than workers who used commonly available information sources. The aim of this study was to answer the following question: is there a difference in the rate of correct answers to questions about OSH between workers who use expert advice through an online expert network and workers who use common information sources?

## Methods

### Study Design

In a quasi-experimental study, we compared the rate of correct answers formulated by a group of workers who used an online expert network with that of a group of workers who used common information sources.

### Online Network on OSH: ArboAntwoord

**Figure 1:**
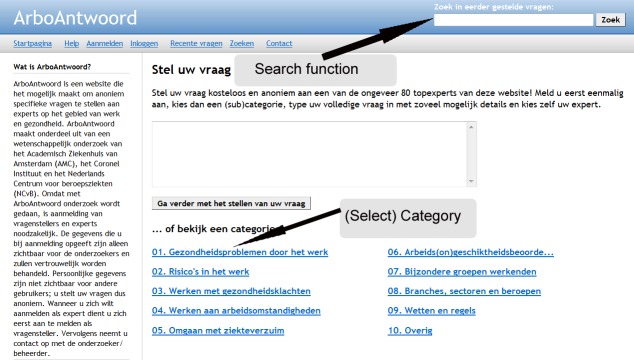
Screenshot of the ArboAntwoord home page, where a questioner can select a question category and use a search function to find stored questions and answers.

**Figure 2:**
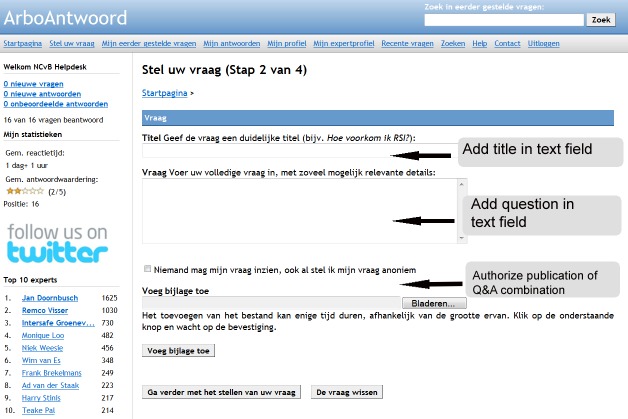
Screenshot of the webpage in a subcategory, where workers actually pose their question, give the question a title, and authorize publication.

**Figure 3:**
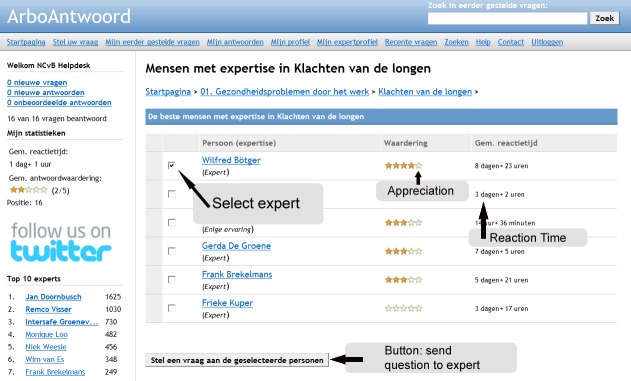
Screenshot of the webpage in a subcategory, where a questioner can select one or more experts. The webpage also includes the experts’ mean reaction time and appreciation and a button to send the question.

**Figure 4:**
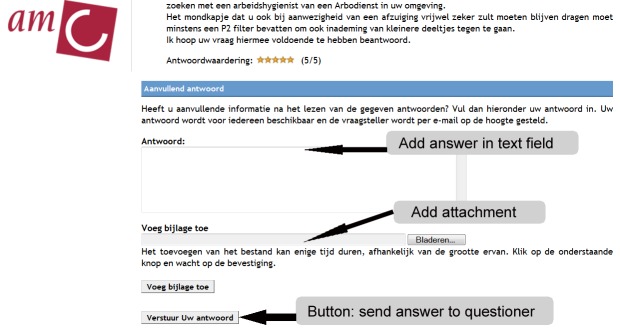
Screenshot of the webpage in a subcategory, where an expert can provide his or her answer, with or without an attachment. The page includes a button to send the question.

**Figure 5:**
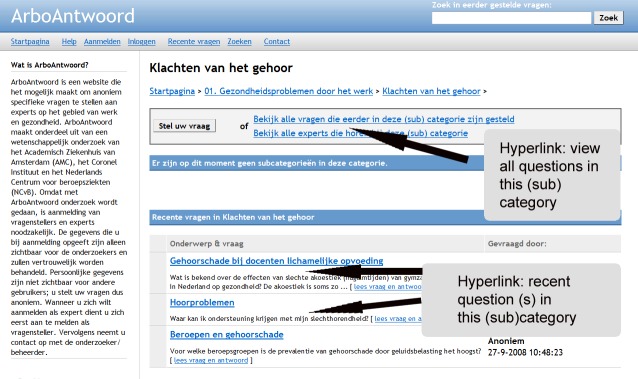
Screenshot of the webpage in a subcategory that provides workers with a hyperlink to view stored and recently asked questions and answers in that subcategory.

ArboAntwoord is an experimental, free-of-charge facility for workers with OSH questions [23,24]. The network was launched by means of a small-scale campaign in October 1, 2008. The home page of ArboAntwoord comprises several main categories of leading OSH topics (Figure 1). After registration, a worker can pose his or her question directly in the designated text field on the home page, or he or she can use the button “ask your question” that is presented in all subcategories. Both possibilities lead to a webpage in which the question must be given a title and the questioner must prohibit or authorize the publication of the question (Figure 2). After formulating his or her question, the worker needs to select one or more experts in that subcategory (Figure 3). Every expert is indexed to the subcategory that corresponds to his or her expertise. Appreciation scores expressed by earlier questioners and mean reaction time to previously answered questions are provided to facilitate questioners’ choosing an expert. Questioners may choose more than one expert. To notify the selected expert about his or her asked question, the page provides a “send question to the expert” button. With this button the selected expert will receive an email notification with a direct hyperlink to the question. Here the expert is also provided a main text field where he or she can also add an attachment when wanted (Figure 4). An expert can notify the questioner about his or her answer by using a “send answer to the questioner” button. All stored questions and answers are published and can be searched by other users when authorized by the questioner and the moderator. When required, experts can react to published questions and answers. Eligible questions and answers are stored and made accessible to other questioners in a searchable database after moderation and after informed consent by the questioner (Figure 5).

A steady network of about 80 experts participate in the network. All experts are invited and/or accepted to participate if they meet all of the following criteria: (1) working in a university, or a commercial OSH expert center or OSH organization operating on a national level, (2) having (inter)national expertise on a specific OSH topic, (3) having at least 5 years of experience on this topic, and (4) participating in at least one knowledge-dissemination activity such as authorship of scientific articles or participation in an expert committee. The professions of the experts vary: occupational physicians, hygienists, safety workers, health scientists, psychologists, neuropsychologists, and experts in OSH law and regulations. All ArboAntwoord experts participate on personal title and on voluntarily basis. Discussion among experts is not common, although about a third of the questions are answered by more than one expert.

### Intervention Group

The intervention group was instructed to use the online network ArboAntwoord for solving 2 OSH questions that were provided by the researchers. As we did not have the opportunity to observe and log their use of the online network directly, we incorporated the 2 OSH questions into paper logs. These logs were mailed to the participants. A log included a question, a field to provide an answer, and several secondary outcome-related questions. A short additional questionnaire for information on background characteristics such as gender, age, educational level, work role, company size, and company sector was sent with the logs. We mentioned that ArboAntwoord was an experimental, free-of-charge online network of experts answering workers’ OSH questions, and we explained that they had to register on the website. Participants were instructed to pose a question in the (subcategory) they thought was the most convenient. The participants were requested to provide a clear and complete answer. Lastly, participants were asked to return the completed logs to the researchers in the return envelop provided. They could also deliver the logs with the start of the course, explained below. Participants had a maximum of 3 weeks to fill in the logs. As an incentive, the participants were promised a memory stick with useful OSH information.

### Control Group

The control group can be interpreted as a care-as-usual group, using the information-seeking strategies and sources of workers with OSH questions in daily practice. Similar to the intervention group, the control group was sent 2 OSH questions that were incorporated into paper logs. These logs included a question, a space to provide the answer, and several secondary outcome-related questions. The logs of the controls instructed them that “You may use all (types of) information sources available to you to solve the questions.” Additionally, we asked them where they looked for information. For this question we provided 4 answer categories: (1) Internet using Google, (2) written sources (ie, magazines and books), (3) experts or specialists, and (4) other sources of information. In addition to this question, controls were requested to note the source that provided the most relevant information. Lastly, we asked the question “How much time in minutes did you spent seeking the information?” The participants were requested to provide a clear and complete answer. They were asked to fill in the logs and return these to the researchers. In total, participants got about 3 weeks to finish the logs. As an incentive the participants were promised a memory stick with useful OSH information.

### Participants

Because we needed a motivated group of workers for this study, we decided to recruit workers who were enrolled to take part in a course for OSH supervisor (in Dutch: preventiemedewerker). An OSH supervisor is a common worker who is responsible for recognizing and suggesting basic solutions for OSH risks in a company. In general, short courses lasting 1–2 days on basic OSH issues are provided to educate and train new supervisors. Although workers enrolled in these courses probably have a higher than average interest in OSH, we know from training reports that more than 80% of the workers start without any substantial knowledge of OSH. Therefore, before the start of a course, we consider enrolled workers to be OSH interested, but not OSH educated. We approached all enlisted course participants from 2 training organizations during 2010 (N = 192). There were no important differences in the content of the course programs. Workers enrolled in the OSH supervisor courses prior to July 2010 were allocated to a control group (courses 1–16; n = 105). All workers enrolled in a course starting in August 2010 or later that year were assigned to an intervention group (courses 17–29; n = 87).

### Data Collection

Both groups were offered 2 questions from a pool of 16 standardized OSH questions. As the difficulty and the topic of the question may affect the likelihood of finding a correct answer, we included these aspects in the construction of the question pool. For question difficulty, we distinguished *simple* information or knowledge questions and *complex* interpretation or advice questions. A simple question was defined as an OSH question that could be answered directly by one specific piece of information or advice. A complex question was defined as a question that could only be answered by interpreting and combining several pieces of information, often accounting for contextual aspects. We formulated 8 simple and 8 complex questions. We further distinguished 2 questions topics. Of the 16 questions, 5 considered OSH laws and regulations (eg, Are safety shoes obligatory for workers in an army storage depot?). The remaining 11 questions were about actual OSH issues, such as causes and risks factors for work-related health and safety (eg, Is radiation a risk for pregnant magnetic resonance imaging workers?), diagnosis (eg, What are the diagnostic criteria for designating posttraumatic stress disorder as an occupational disease?), interventions in occupational safety or health (eg, What is an effective intervention for occupational dermatitis?), or social mapping (eg, Where can I find the best expert on chronic low back pain rehabilitation?).

To increase the ecological validity (the extent to which research emulates the real world) of the 16 OSH questions to be selected, we randomly selected 12 questions with answers from the ArboAntwoord database [25]. Additionally, 4 simple questions were formulated by the researchers based on information provided in the OSH supervisor course handbooks, and these questions were added to the pool. For the random selection of the 12 ArboAntwoord questions, all 319 questions in the ArboAntwoord database were first stratified by 2 researchers independently (MR and AF) in 4 categories based on difficulty (simple or complex) and topic (OSH law and regulations, or OSH content). Subsequently, 2 simple OSH law and regulation questions, 3 complex OSH law and regulation questions, 6 simple OSH content questions, and 5 complex OSH content questions were used in this study. An example of a difficult OSH content question is “Are the glass fibers or dust released after the crushing, cutting or fragmentation of (car) windows in the open air hazardous to my health? What can be done to prevent hazards?” We observed that the structure of 8 questions included 2 components—that is, these questions were actually composed of 2 questions. This corresponds to our experience with questions from practice, where workers often seem to have a concern about an OSH risk and wonder about a possible solution. The 16 questions are included in Multimedia Appendix 1.

We randomly assigned 2 questions to each participant and sent them out about 3 weeks before the start of their OSH supervisor course, ensuring that all participants received 1 easy and 1 complex question (to ensure they would not be discouraged by getting 2 complex questions). Based on the level of question difficulty, an automatically created randomization list was used with 56 possible combinations (8 simple × 8 – 1 complex questions) for both the intervention and control groups. Because we anticipated about 200 course participants and a response rate of 50%, we expected that every question would be answered about 6 times in the control group and 6 times in the intervention group. Although participants in the intervention group posed a question directly to an ArboAntwoord expert, the system moderator (MR) always provided questioners with the original answers to 12 randomly selected OSH questions to prevent unnecessary use of expert time and to avert possible learning effects resulting from answering the same question more than once. For the 4 self-formulated OSH questions we used the first answer provided by an expert in our experiment.

### Primary Outcome and Scoring Procedure

To define the main study outcome parameter *answer correctness* we used the experiences and approaches of evidence-based practice [9,10]. Evidence-based practice is a strategy for clinical decision making that involves the integration of the patients’ needs and context, the expertise of professionals, and the best available research evidence. In this study, we define a correct answer as “an answer that accounts for the context of the question(er) and corresponds with conclusions or recommendations of the best available evidence”. Two steps were needed to assess whether provided answers were correct.

First, 16 standardized correct *model* answers were constructed with the help of 2 reviewers with expertise on the question topics. A total of 10 reviewers were internal experts who also participated in the ArboAntwoord network, and 13 external reviewers were recruited for the review process. Internal experts could not review their own answers. All reviewers were provided with a draft model answer formulated by the research team. The reviewers were requested to report whether the draft model answer was in complete, partial, or no agreement with the conclusions or recommendations from the best available evidence. Similarly to the evidence-based practice method, reviewers were asked to consider 4 levels of evidence by means of an evidence literature search looking for evidence-based guidelines, reviews, or scientific research articles, or using their own professional expertise, in that order. If evidence from the highest level was not available, we requested the expert to move on to the next best level of available evidence. Because these levels of evidence do not apply to OSH law and regulation questions, reviewers of draft answers on this topic were requested to first regard laws and regulations, policy, jurisdictions, or standards, followed by their own professional expertise. All additional evidence and comments provided by both reviewers were included in the final versions of the standardized correct model answers.

Second, we developed a scoring system defining correctness for all 16 standardized model answers separately based on the essential aspects required for a correct answer (see Multimedia Appendix 1). The scoring system consisted of an answer correctness score between 0 and 4. The essential aspects were directly dependent on the nature of the particular OSH question. For example, questions sometimes asked for confirmation or proof, for conditions under which an OSH situation holds, for a specific location, for possible risks or solutions, or for a combination of these. To verify whether a participant finally reached the correct answer conclusion to the main question, we included this as one of the essential aspects in all 16 model answers. This aspect can be considered as the correctness of an answer’s main conclusion (yes, no, or possibly). The question about the health hazards of glass fibers and dust discussed earlier in the Methods was scored as follows. The main conclusion, “Yes, this could possibly be hazardous to health,” was given 1 point. Another point was provided when the answer mentioned something like “Depending on glass (particle) type and exposure.” Two more points were awarded when a security measure were given. As in health care in general, there is sometimes some variation or interpretation for what constitutes a correct, evidence-based answer. This especially holds true for the questions related to OSH content. For all questions some room was created to account for this variation and interpretation issue (Multimedia Appendix 1). Based on the scoring system, all participants’ answers were scored and compared with the model answers by 2 raters. The first rater was a medical student unrelated to this study. The second rater, MR, checked the answer scores of the first rater. Both raters were blinded to the group (intervention or control) to which the answers belonged.

### Secondary Outcomes

We assessed 2 secondary outcomes: (1) the experienced quality of the information source used: whether the source was usable or easy to use, and how easy it was to learn to use, and (2) the experienced quality of the information obtained: whether the information was complete, applicable, and reliable, and how satisfied the participant was with the information. All response categories to these questions were based on 7-point Likert scales (ranging from completely disagree to completely agree).

### Data Analysis

We described most outcomes by means of descriptive analysis. Analyses were performed with SPSS version 17.0 (IBM Corporation, Somers, NY, USA). To establish any group differences in background characteristics that required adjustment in further analysis, we first applied the chi-square test for dichotomous or nominal variables, and the Yates and Cochrane test for ordinal variables (*P* < .05).

The correctness of the participants’ answers was analyzed in 2 ways. First, we verified whether the answers given were sufficiently correct by dichotomizing the 4-point answer scores (0–2 points = insufficiently evidence based; 3–4 = sufficiently evidence based). We considered using the ordinal data, but we observed that the distribution was skewed. Thus, dichotomization seemed the best option without giving away a lot of information. Second, we looked at the correct main conclusions of the answers. Possible group differences between the intervention and control groups regarding the prevalence of correct answers and of correct main conclusions were analyzed with the chi-square test (*P* < .05). Because we found no differences between the groups in terms of question difficulty, question topic, or background characteristics (*P* > .05), we used binary logistic regression analysis only to establish possible interaction effects of these factors on the main outcome(s) (*P* < .05). We stratified the effect of group type on the number of correct answers by question difficulty, question topic, question structure, and background characteristics. We used the chi-square test for dichotomous and nominal variables, and the Yates and Cochrane test for the ordinal background characteristics (*P* < .05). To describe the strength of associations, we used rate differences. In this study, rate difference is probably the most appropriate measure because it describes the absolute change in the rate of, for example, correct answers attributable to the intervention.

Potential differences between the groups regarding the 2 secondary outcomes (the experienced quality of the information source used and the information it provided) were analyzed with the Wilcoxon rank sum test (*P* < .05). Because we observed moderate to high Spearman correlations (*r* = .45–.65) and good internal consistency (Cronbach alpha = .74) between the 4 items on the experienced quality of the source used, these 4 items were processed into a single-item factor by calculating mean scores. We observed no high correlations between the 4 items on information quality (*r* < .40).

In the control group we also applied the Wilcoxon rank sum test to determine the effect of information-seeking time and experienced information quality or quality of the information source used on providing correct answers.

## Results

### Group Characteristics

Overall, 47 of the 87 (54%) workers assigned to the intervention group agreed to participate in the study compared with 62 of the 105 (59%) in the control group. This resulted in 94 answers in the intervention and 124 answers in the control group. In total 110 of the 124 (89%) questions in the control group were answered with information obtained online, 9 (7%) with information from written sources (ie, magazines, books), and 5 (4%) with advice from experts or specialists. Because removing questions that were answered with written information or expert advice did not change any of the outcomes, these questions were preserved in further analysis. The median information-seeking time in the control group was 10 minutes per question (interquartile range: 5–20 minutes), and this time in minutes was not comparable with the time in days the intervention group had to wait for their answer of the ArboAntwoord experts. We did not observe any significant group differences in background characteristics (*P* > .05) (Table 1). Young participants between the age of 15 and 24 years were not represented in either group.

**Table 1:** Background characteristics of the intervention group (n = 47) and the control group (n = 62)

Characteristic		Intervention group (online network ArboAntwoord)		Control group (common information sources)		
		n	%	n	%	
**Gender**						
	Female	28	60	33	53	
	Male	19	40	29	47	
**Age group (years)**						
	15–24	0	0	0	0	
	25–34	17	36	14	23	
	35–44	12	25	22	35	
	45–54	13	28	20	32	
	≥55	5	11	6	10	
**Educational level**						
	Low	11	23	13	21	
	Intermediate	17	36	21	34	
	High	19	40	28	45	
**Role**						
	Worker	32	68	42	68	
	Employer/manager	8	17	13	21	
	OSH^a^ (semi)professional	7	15	7	11	
**Company size**						
	Small	19	40	27	44	
	Medium	16	34	18	29	
	Large	12	26	17	27	
**Company sector**						
	Agriculture and fishery	2	4	3	5	
	Industry	13	28	12	19	
	Construction industry	4	10	6	10	
	Trade	8	17	11	17	
	Transport and communication	1	2	3	5	
	Financial services	1	2	1	2	
	Business services	8	17	7	11	
	Public policy or civil service	3	6	3	5	
	Education	1	2	3	5	
	Health care	3	6	7	11	
	Culture and other services	3	6	6	10	
**Self-rated Internet and computer experience**						
	Relatively inexperienced	8	17	16		26
	Relatively experienced	39	83	46		74

^a^ Occupational safety and health.

We observed no statistical group differences in the distribution of simple or complex questions (χ^2^_1_ = 0; *P* = .9) and OSH legislation or OSH content questions (χ^2^_1_ = 0; *P* = .9). Nevertheless, we observed a discrepancy in the distribution of the answers to 2 of the OSH questions. Question 7 was answered once in the intervention group and 4 times in the control group, and question 13 was not answered at all in the intervention group and was answered 8 times in the control group. Because removing these 2 questions did not change any of the outcomes, both were preserved in further analysis. All other questions were answered between 4 and 10 times in both groups.

### Answer Correctness

In total, 58 of the 94 (62%) answers of the intervention group were rated correct, compared with 24 of the 124 (19%) answers for the control group. A significant difference with a rate difference of 43% was observed (95% CI 30%–54%) (Table 2). The use of the online expert network ArboAntwoord had a positive effect on providing correct answers, and the effect was identical for answers to simple or complex questions, for answers to questions related to OSH law and regulations or OSH content, and for answers to single or double questions (Table 2). Stratification by background characteristics consistently showed similar differences in the distribution of evidence-based answers in favor of the intervention group, with rate differences ranging from 33% to 66% (Table 2). Only Internet use significantly interacted with the effect of intervention on answer correctness: using the online network ArboAntwoord for providing correct answers was found to be even more beneficial for relatively inexperienced Internet and computer users (Wald χ^2^_1_ = 3.9; *P* = .048).

In the intervention group, answers to questions about OSH law and regulations were significantly more often correct than questions about OSH content (χ^2^_1_ = 7.9; *P* = .01). This same trend was observed in the control group (χ^2^_1_ = 3.2; *P* = .07). Furthermore, within the control group we found that correct answers were significantly less often provided by men than by women (χ^2^_1_ = 5.7; *P* = .02), with a similar trend for gender in the intervention group (χ^2^_1_ = 3.7; *P* = .06). Spending more time seeking information did not affect the likelihood of finding a correct answer (*Z* = –0.18, *P* = .9).

Finally, we analyzed a subgroup on the one essential aspect that was similar for all answers: the correctness of the main conclusion (yes, no, or possibly). In total, 85 of the 94 (90%) main conclusions in the intervention group were correct compared with 75 of the 124 (61%) conclusions for the control group. A significant difference with a rate difference of 29% was found (95% CI 19%–40%) (Table 3). This positive effect in favor of the intervention was identical for answers to simple and complex questions, for answers to questions related to OSH law and regulations and OSH content, and for answers to single and double questions.

**Table 2:** Rates of correct answers in the intervention group (n = 94 answers) compared with the control group (n = 124 answers) stratified by question difficulty, question topic, question structure, and background characteristics

		Intervention group (online network ArboAntwoord)		Control group (common information sources)			Intervention vs control group	
		n/N	%	n/N	%		RD%^a^	95% CI^b^
Total (all questions)		58/94	62	24/124	19		43	30–54
**Question difficulty**								
	Simple	25/47	53	12/63	19		34	16–50
	Complex	33/47	70	12/61	20		50	33–65
**Question topic**								
	OSH^c^ law and regulations	24/29	83	11/38	29		54	31–71
	OSH content	34/65	52	13/86	15		37	22–51
**Question structure**								
	Single	18/31	58	10/50	20		38	17–57
	Double	40/63	63	14/74	19		44	29–58
**Gender**								
	Female	39/56	70	18/66	27		43	25–57
	Male	19/38	50	6/58	10		40	22–56
**Age group (years)**								
	15–24	NA^d^		NA			NA	
	25–34	22/34	65	6/28	21		44	19–63
	35–44	15/24	63	10/44	23		40	15–60
	45–54	16/26	62	6/40	15		47	23–66
	≥55	5/10	50	2/12	17		33	–7 to 66
**Educational level**								
	Low	14/22	64	4/26	15		49	21–69
	Intermediate	22/34	65	9/42	21		44	21–61
	High	22/38	58	11/56	20		38	19–56
**Role**								
	Worker	40/64	63	20/84	24		39	23–53
	Employer/manager	7/16	44	1/26	4		40	16–64
	OSH (semi)professional	11/14	79	3/14	21		58	20–80
**Company size**								
	Small	22/38	58	9/54	17		41	22–58
	Medium	16/32	50	5/36	14		36	14–55
	Large	20/24	83	10/34	29		54	29–72
**Self-rated Internet and computer experience**								
	Inexperienced	12/16	75	3/32		9	66	38–8
	Experienced	46/78	59	21/92		23	36	22–49

^a^ Rate difference.

^b^ Confidence interval.

^c^ Occupational safety and health.

^d^ Not applicable.

**Table 3:** Rates of correct main conclusions of answers in the intervention group (n = 94 answers) compared with the control group (n = 124 answers) stratified by question difficulty topic and structure

		Intervention group (online network ArboAntwoord)		Control group (common information sources)		Intervention vs control group	
		n/N	%	n/N	%	RD%^a^	95% CI^b^
Total (all questions)		85/94	90	75/124	61	29	19–40
**Question difficulty**							
	Simple	41/47	87	40/63	64	23	7–38
	Complex	44/47	94	35/61	57	37	21–50
**Question topic**							
	OSH^a^ law and regulations	28/29	97	26/38	68	29	11–45
	OSH content	57/65	88	49/86	57	31	17–43
**Question structure**							
	Single	31/31	100	31/50	62	38	26–52
	Double	54/63	86	44/74	60	26	11–40

^a^ Rate difference.

^b^ Confidence interval.

^c^ Occupational safety and health.

### Experienced Quality of the Information (Sources) Used

On average, the online network ArboAntwoord was rated of higher quality than common information sources, with mean scores of 5.8 (interquartile range: 5.5–6.3) and 5.2 (interquartile range: 4.4–6.0), respectively (*Z* = –3.5, *P* < .001). Participants in the intervention group experienced the completeness of the received information to be significantly higher (Z = –2.6, P = .01) and were significantly more satisfied with the received information (Z = –2.3, P = .03) than participants in the control group (Table 4). Notably, judging the quality of the information seemed to be difficult for the participants. Within both the intervention and the control group, we did not find a significant difference in the experienced information quality scores between workers who provided correct answers and those who did not (within the intervention group: information completeness *Z* = –0.9, *P* = .4, applicability *Z* = –1.0, *P* = .3, and credibility *Z* = –1.5, *P* = .1; within the control group: information completeness *Z* = –0.8, *P* = .4, applicability *Z* = –1.3, *P* = .2, and credibility *Z* = –0.6, *P* = .5). This finding corresponds to the comparably high applicability and credibility scores found for both ArboAntwoord and the common information sources.

**Table 4:** Comparison between the intervention group (n = 94 answers) and the control group (n = 124 answers) regarding experienced information completeness, applicability, credibility, and satisfaction with the information

Experienced information quality	Intervention group (online network ArboAntwoord)		Control group (common information sources)		Intervention vs control group *Z*	Intervention vs control group *P* value
	Mean	IQR^a^	Mean	IQR		
Completeness	5.4	5.0–6.0	4.7	3.0–6.0	–2.6	.01
Applicability	5.5	5.0–6.0	5.3	5.0–6.0	–1.2	.2
Credibility	5.4	4.0–6.0	5.4	5.0–6.0	–0.05	.9
Satisfaction	5.6	6.0–6.0	5.0	4.8–6.0	–2.3	.03

^a^ Interquartile range.

## Discussion

### Principal Results

Our findings show that the rate of correct answers to OSH questions provided by workers who used expert advice obtained from an online network was significantly higher than the rate of correct answers provided by workers who used common information sources. When workers used their common information sources (in 90% of the cases, these were informational websites found through Google), only 19% of the answers were correct. The rate of correct answers was 62% for workers using the online expert network ArboAntwoord, which is significantly higher. This difference was found for answers to simple and complex questions, for answers to questions about OSH law and regulations and about OSH content, and for answers to single and double questions. Answer correctness rates in both groups increased to 90% and 61% when we analyzed only the correctness of the main conclusion of the answers. Overall, workers who used ArboAntwoord were more satisfied with information that they received, and they experienced the information as more complete than workers who used common information sources. Nevertheless, the perceived information quality scores were relatively high in both groups. Remarkably, within both the experimental and control groups, workers who provided incorrect answers believed the information that they used to be as credible, complete, and applicable as did workers who provided correct answers. Workers appear to be unable to judge the quality of the information they find.

### Comparison with Prior Work

To our knowledge, this is one of the first studies evaluating whether and how workers can find correct evidence-based answers to OSH questions. So far, most studies on answering OSH questions focus on OSH professionals and their use of evidence-based practice strategies [6,7,18,19,26]. Our findings clearly demonstrate that workers provide correct answers more often with the support of expert advice than with common information sources*.* Because we checked that all information necessary to answer the 16 questions correctly was available on the Web, our findings might be partly explained by the fact that workers have limited search skills and seem unable to judge important qualities of the information they find. Consequently, workers who wrongly judge the credibility, completeness, and applicability of information as high are likely to provide an incorrect answer. This especially holds true for participants in the control group, who often used online information found with Google. In several studies, non-health professionals have been found to use too few search terms and to select only one of the first few results displayed by a search engine [27-29]. Moreover, the quality of information found with these search engines has been shown to vary [14,16,30-32]. Consequently, using search engines such as Google does not always result in finding correct evidence-based answers. Thiele et al [33] demonstrated that medical students, resident physicians, and attending physicians provided only about 65% correct answers to 8 anesthesia and critical care-based clinical questions using Google. Similarly, Kingsley et al [34] found that only 25% of first-year dental students provided correct answers to several fundamental biomedical questions when using Google. In a study by Tang and Ng [17], several samples of diagnostic cases were selected from the *New England Journal of Medicine* that were subsequently googled for a diagnosis. The searches revealed a correct diagnosis in only 58% of the cases. Moreover, Kortum et al [35] showed that nonprofessionals (high school students) often provide incorrect answers to health questions when using the Internet. Consistently with our results, they concluded that difficulties in distinguishing trustworthy from untrustworthy medical information resulted in these incorrect answers. In sum, similar to the concept of health literacy [13], OSH literacy corresponding to “the cognitive and social skills which determine the motivation and the ability of an individual to gain access to, understand and use information in ways that promote and maintain good occupational safety and health” seems to influence workers’ ability to find correct answers to OSH questions. It would be worthwhile to further explore OSH literacy and its role in finding correct answers effectively.

The higher rate of correct answers in the intervention group was probably further amplified by the high proficiency of the experts associated with ArboAntwoord. They are leading national experts on specific OSH topics and are familiar with finding, selecting, appraising, and applying evidence-based information. Nevertheless, even these experts did not always provide a correct answer, which is in accordance with the findings of Schaafsma et al [36], who stated that caution is required when relying blindly on expert advice. Again, it is possible that participants sometimes did not understand or interpret the information of the experts correctly. Another possible explanation is that experts were hindered by a lack of time in answering questions thoroughly and performing evidence searches when needed [26]. Moreover, the experts in ArboAntwoord participated voluntarily and were not paid. In commercial, nonhealth-related networks, (perceived) answer quality was shown to increase when users were paid to provide answers [37]. Possibly, to further increase answer correctness, experts could be given small incentives and more thorough instruction in how to answer questions correctly.

Subgroup analysis resulted in additional interesting findings. Within the intervention and control groups, we observed that the rate of correct main conclusions was much higher than the rate of correct answers in general. It is possible that workers are often able to provide a sort of “logical” conclusion based on deduction, observation of current practices, common sense about moral responsibility, or even implicit knowledge. Additionally, the a priori chance to provide a correct conclusion is 33% (yes, no, or possibly), which may also partly explain why, in both groups, the rate of correct conclusions was higher than the rate of correct answers in general.

In both the intervention and control groups, the rate of correct answers to questions about OSH law and regulations was higher than that of answers to questions about OSH content. Apparently, OSH content questions are more difficult to answer than questions about OSH legislation for both questioners and experts. Possibly, the (poor) formulation of the OSH content questions might have made them more difficult to answer. Finally, in both the control and intervention groups, we found a trend that women seemed to outperform men. It is possible that men feel less obligated, motivated, or aroused to find correct answers to the questions, or they may have less efficient learning styles (including information-seeking strategies) that affect the effort of seeking information [38,39].

Two expected effects could not be established in our analyses. We expected that for complex questions the rate of correct answers would be significantly lower than for simple questions, especially in the control group. We presumed that expert advice would be particularly necessary for the complex questions. Our findings did not corroborate these expectations. Our hypothesis may have been incorrect, or it is possible that our simple questions were not actually very simple, or our complex questions were not actually very complex. Finally, we expected that information-seeking time would influence the rate of correct answers in the control group: that spending more time seeking information would have a positive influence on this rate. However, this difference could not be established. Again, because workers often seem to misjudge information quality, they believe that spending more time on information seeking is unnecessary. Another explanation might be that self-reporting of the time spent seeking information is subject to social desirability bias. Estimating the time required might therefore be less reliable than actually observing and timing the information-seeking process.

### Strengths and Limitations

This study has several apparent strengths. The use of specific OSH questions from practice increases the ecological validity of our study. The stratified assignment of questions, the blinding of raters regarding the group to which the answers belonged, and the quasi-experimental design improved the quality of the study. Our study also has methodological limitations. The selection of workers who were planning to take part in an OSH supervisor course limits the generalizability to all workers. In addition, workers younger than 25 years were not represented in either group. As we believe that our sample may have been more motivated because of their proven OSH interest, the low rate of correct answers in the control group may be an overestimation.

Furthermore, although selecting OSH questions from ArboAntwoord may have increased the ecological validity, it also introduced a limitation. Participants probably did not personally relate to these specific OSH questions, and this might have caused participants to be less motivated to find an answer. This effect could be more apparent in the control group, who had to find an answer to these questions on their own. An alternative study design, letting participants bring in their own OSH question, may increase workers’ understanding and commitment with the question, and as a consequence the efforts spent answering it. A disadvantage of this design is the potentially poor comparability of the outcomes between the two groups. The selection and composition of the questions might constitute another limitation. Our distinction between simple and complex questions can be questioned, as this was based on our personal estimation of whether an answer needed the combination and interpretation of information (complex). In retrospect, almost all our questions may be regarded as fairly complex, which might have caused the rate of correct answers to be lower than in daily practice. Furthermore, in view of classic evidence-based practice methods, at least several of the selected OSH questions seem poorly formulated. The accurate formulation of a clinical question is often mentioned as one of the most important skills required for evidence-based practice [9,10]. Several of our questions comprise more than one issue, do not address a specific target group, do not define a clear outcome, or do not take into account important contextual factors. Consequently, a poorly formulated question is more difficult to answer correctly, especially for participants in the control group, who had to interpret the question themselves. Moreover, this might also have influenced what we defined as a correct model answer, especially because for several questions the highest available level of evidence was the experts’ (reviewers’) opinion. In these cases, expert reviewers other than those involved in this study might have had a different opinion on what constitutes a correct evidence-based answer. In this line, the design and use of our model answers require consideration. For example, in this study we decided to dichotomize our ordinal data because of a skewed distribution. This may have caused some loss of information. Future studies may consider using continuous correctness scores. Lastly, our way of data collection may constitute a limitation. Participants had to answer the questions and complete the paper logs at home or at work. It might have been better to let participants search for information in the lab, where we could have videotaped them and assessed computer log files. However, the advantage of a field test is that it represents the real-life situation better than a laboratory experiment and that it is possible to study search strategies other than online ones.

### Conclusions and Implications

Workers are often unable to find correct evidence-based answers to OSH questions when using common information sources, generally informational websites. The limited experience of workers with finding high-quality information seems to play an important role in finding correct answers; workers seem to be unable to judge the credibility, completeness, and applicability of the OSH information they find. Future research should explore workers’ OSH information-seeking skills, their appraisal of information quality, and their ability to apply the obtained information to solve their question.

In addition to common information sources, other strategies and sources are required to assist workers in answering their OSH questions and to overcome difficulties in finding high-quality information. Expert advice provided through an online expert network (ArboAntwoord) can increase the rate of correct answers substantially, especially when focusing on the correct main conclusions. This purpose might also be facilitated by educational strategies such as short custom-made evidence-based practice courses for workers and managers or their representatives, or decision-support tools, or by providing accreditation to high-quality information. Future research could further establish the effectiveness of these new strategies.

Lastly, the identified difficulties with finding, appraising, and applying health-related information is not unique to workers. It is also relevant to other non-health professionals seeking health information, such as people in the general population or patients. Our findings on the potential value of online expert networks and expert facilities in general seem also applicable to other groups of people seeking answers to their health questions, albeit dependent on the quality of the knowledge infrastructures built around specific health topics (eg, asthma, cancer, or schizophrenia). Future research may focus on the impact of similar expert facilities in other health-related fields.

## Multimedia Appendix 1

The 16 occupational safety and health (OSH) questions and their standardized correct model answers.

## Abbreviations

CI: confidence interval
OSH: occupational safety and health
