# Supplementary material for: Comparing the Use of an Online Expert Health Network against Common Information Sources to Answer Health Questions
Source: J Med Internet Res. 2012 Feb 2;14(1):e9. doi: 10.2196/jmir.1886 (PMC3374530; doi:10.2196/jmir.1886)
Supplement: Supplementary file 1 [file jmir_v14i1e9_app1.pdf]

Question number	Question<br>Question difficulty: simple or complex (1,2); Question topic: OSH law and regulations or OSH content (a,b); Questions structure: single question or double question (I,II)	Essential aspects needed for a correct answer including a main conclusion for the main question<br>Correctness answer: 0 - 4 points
1.	I work with a contractor. Currently we are renovating an old factory with a large group of demolishers, carpenters, electricians and plumbers. The building seems quite dangerous. In many places there seems to be a danger of collapsing, the floors are unstable and wet. Basically I (and some colleagues) do not want to work in this building! Can I decide to stop working on this site? How can I handle this? (1aII)	– Yes, you may decide to stop working on this site: 1 point *[main conclusion]*<br>– The law prescribes that a worker may quit working in dangerous situations (in Arbowet Article 29): 1 point<br>– Action: You have to consult with/report to your employer or manager, about the situation and communicate that you want to quit. Possibly, you should also consult with/report to trade union or work council (toolbox meeting): 1 point<br>– Consulting with/reporting to employer or manager is obligatory before quitting (in Arbowet Article 11): 1 point
2.	I work in a large office for a financial company. All day long I work with a desktop computer. I often experience pain in my lower back, and I am not the only one with these complaints. More people complain about this. What are the possible causes of my low back pain? What are the possible solutions? (1bII)	– Possibly, a lack of movement may be the cause / No, it is probable that sitting behind a computer is the cause of your low back pain: 1 point *[main conclusion]*<br>– Other risk factors are more probable (e.g., lifting, carrying, vibrations, age): 1 point<br>– For now, consult your occupational physician, another occupational health professional or your family physician (these individuals can initiate a physical or a workplace examination): 2 points
3.	I work in a garage as a mechanic. Many devices make a lot of noise (drilling, etc.). I myself feel that I am starting to become a bit deaf. When is noise hazardous for my hearing? And what may offer me protection against hearing loss? (1bII)	– Yes, noise is hazardous if exposure is higher than 80 decibels 8 hours per day for 40 years: 2 points (mention 2 of 3) / 1 point (mention 1) *[main conclusion]*<br>– Protective equipment – a. alleviate the source of the noise b. ear plugs or other hearing protectors: 2 points (mention at least 1)
4.	I own a large living-sleeping furniture store. I employ about 25 people. We sell beds, floors and window decorations. We believe there are several occupational risks. I want to execute an investigation and evaluation of health and safety risks (Dutch: RI&E). Do you know if and where a recognized RI&E for small or medium enterprises exists? Or one even more specific to our trade? (1bII)	– Yes, several (specific) RI&Es exist: 1 point *[main conclusion]*<br>– Name of, or reference to, this RI&E: 2 points<br>– Attention! This RI&E should be modified to your specific business and/or quality is sometimes poor: 1 point
5.	Several people in our company have questions about the potential danger of toners from photocopiers and printers. I try to answer these questions as an OSH supervisor. It is not easy to find correct and relevant information on this topic. Are there any risks/hazards to health? What are the risks/hazards? (1bI)	– Yes, there are health risks/hazards: 1 point *[main conclusion]*<br>– Risk for allergic reaction to skin or lungs (toner disease): 1 point<br>– Probably no other health risks/hazards: 1 point<br>– Risk depends on exposure (time, duration) and type of material (oxidative compounds, solvents, fine dust: 1 point (mention at least one)

6.	I am looking for a booster seat for a chair so that children (6-10 years) in a treatment group can sit at the dinner table (I feed them). It is a treatment group for children and youth within the mental health care system. This booster should be safe and comply with legal standards. Higher chairs are not an option because personnel, parents and young people also have to use them. Where can I find a similar booster seat? (1bI)	– Yes, these booster seats exists: 1 point *[main conclusion]*<br>– A booster seat may not be the safest option/another solution may be better: 1 point<br>– Significant use/safety tip for the booster seat: 1 point (mention at least one)<br>– Name/address where booster seat can be obtained: 1 point
7.	Is there an RI&E for work on pumping stations (rain water/sewage)? Who can help me with an RI&E for working on water supply pumping stations? (1bI)	– Yes, several (specific) RI&Es exist: 1 point *[main conclusion]*<br>– Name of or reference to this RI&E: 2 points<br>– Attention! This RI&E or should be modified to your specific business and/or quality is sometimes poor: 1 point
8.	Working in our packaging department means working with forklifts, lifting pallets and boxes, etc. Most people walk on normal shoes, some even in sandals. I have addressed the manager several times about this issue. In my opinion, we should be wearing safety shoes. Is wearing safety shoes obligatory? (2aI)	– Yes, safety shoes are obligatory: 2 points *[main conclusion]*<br>– Not literally prescribed in Dutch a law/only indirectly prescribed in the law (Arbowet Article 11, Article 5 or chapter 8 Arbobesluit): 1 point<br>– Reasoning: the employer is always responsible and accountable for the health and safety of workers. Therefore, company health and safety policies and/or an RI&E should be in order: 1 point
9.	A chemical disposal car collects the chemical waste from households. Packages are mostly empty and/or well-sealed. In the car, packages are placed in different crates on shelves that each contains a separate risk group. Often the driver does this work alone. Are there any risks when working alone on a chemical disposal car, and how can we best minimize them? (2bII)	– Yes, there are risks: 1 point *[main conclusion]*<br>– Risks are difficult to estimate; it depends on risks associated with the car and the risks associated with the environment à an RI&E should be prepared and approved to identify these risks: 1 point<br>– Solutions: risks should not be larger when working alone than when working with others: 1 point<br>– Solutions: (a) (Think about) setting up a warning system: 1 point, (b) Other possibilities for management of risks:<br>1 point (at least one other plausible solution/measure).
10.	Are the glass fibers or dust released after the crushing, cutting or fragmentation of (car) windows in the open air hazardous to my health? What can be done do prevent hazards? (2bII)	– Yes, that could possibly be hazardous to health (skin, lungs, eyes, probably not cancer): 1 point *[main conclusion]*<br>– Depending on glass (particle) type and exposure. There are many different types of windshields, which are made of various substances. Some of these may be harmful (although there will be no glass fiber released with cutting or crushing glass. These fibers are carcinogenic): 1 point<br>– Security aspects are important: to minimize the risk of a fine dust, a mask, gloves and/or goggles are wise: 2 points

11.	We are in the process of opening an office. I wondered whether the use of laminate flooring has audiological drawbacks and who could measure these? (2bII)	– Yes, laminate can cause noise interference or disturbance: 2 points *[main conclusion]*<br>– This noise is dependent on the following: 1. the characteristics of the room, 2. the sub-floor, 3. the work requirements 4. the types of employees: 1 point (mention at least one)<br>– Measurements: audiology center, acoustical consultancy bureaus or occupational health services have measurement equipment and advisors: 1 point
12.	To me it is clear what an OSH catalogue is [Dutch: arbocatalogus], but I do not know what an OSH manual is [Dutch: arbohandboek]. So, is an OSH catalogue the same as an OSH manual? Or, is it another term for the same thing? (1aI)	– No, an OSH catalogue differs from a OSH manual: 2 points *[main conclusion]*<br>– A catalogue is for a sector (level); a manual is for a company (level): 1 point<br>– A catalogue should be reviewed by the ministry and/or company that is responsible for the safety and health of employees: 1 point
13.	I work in a production laboratory where they produce blood and intravenous (IV) bags. In recent years a number of colleagues have been struck by cancer: breast cancer, kidney cancer and brain tumors. I would not directly say that this is caused by gluing these bags, but it causes many concerns. THF (Tetrahydrofuran) glue is widely used during the production process. In previous years, the glue fumes were extracted by local exhaust ventilation from the ceiling, but in recent years, from the floor. When the bags are glued the smell of the glue can be detected in the air. So the question is as follows: can THF glue cause cancer? (2bI)	– No, THF glue cannot cause cancer/Maybe, THF can cause cancer: only one animal study demonstrated a relationship between THF and cancer: 1 point *[main conclusion]*<br>– It is not on the IARC or Dutch government list of cancer-causing substances: 1 point<br>– Fume extraction by local exhaust ventilation is what makes it less probable that THF can cause cancer: 1 point<br>– The occurrence of different types of cancer within one profession is highly unlikely: 1 point
14.	In our office, there are many complaints of dry eyes, nasal congestion and other similar symptoms. Could this caused by low humidity? I would also like to know how low can/should the humidity be? (2bII)	– Yes, it (1 point) can in extreme cases (<15% - 30% humidity)/No, in principle it cannot: 2 points *[main conclusion]*<br>– Throat, nose and/or voice complaints: 1 point (mention at least one)<br>– The exact range (between 30% and 70%) is not prescribed in any Dutch law/It is only indirectly prescribed in Dutch law: the employer is responsible for the safety and health of employees: 1 point
15.	We have a very small shop. Recently, we started working alone. We work for 8 hours. This often means no pause, no toilet, and it causes an unsafe feeling at night. I do not think this is in correspondence with the OSH laws! Hence the following questions: is this in correspondence with the law? Additionally, how can I respond/react to my supervisor/manager? (2aII)	– No, you may not work 8 hours in a row according to the law: 1 point *[main conclusion]*<br>– The law is clear à after 5.5 hours of work, a fifteen-minute break is mandatory: 1 point<br>– Consult with the employer/manager or work council on what they think of the situation and how it can be improved: 2 points

16.	When is an excavation an enclosed space? [in construction] (2aI)	– Clear and unambiguous definition of a closed space: 4 points *[main conclusion]*<br>OR: two important elements of the definition of a closed space:<br>– Limited accessibility: 2 points<br>– Danger of suffocation, intoxication or poisoning due to collapse (rule: trench deeper than 1 meter): 2 points<br>(mention at least one)
